# Incorporating the effect of the photon spectrum on biomass accumulation of lettuce using a dynamic growth model

**DOI:** 10.3389/fpls.2023.1106576

**Published:** 2023-05-23

**Authors:** Mahyar Abedi, Xu Tan, Eric J. Stallknecht, Erik S. Runkle, James F. Klausner, Michael S. Murillo, André Bénard

**Affiliations:** ^1^ Department of Mechanical Engineering, Michigan State University, East Lansing, MI, United States; ^2^ Department of Horticulture, Michigan State University, East Lansing, MI, United States; ^3^ Department of Computational Mathematics, Science and Engineering, Michigan State University, East Lansing, MI, United States

**Keywords:** plant growth, dynamic modeling, spectral distribution, *Lactuca sativa*, indoor crop production, regression-based modeling, controlled environment agriculture

## Abstract

Cultivation studies in specialty crop optimization utilize models to estimate the fresh and dry mass yield. However, the spectral distribution and photon flux density $\left(\mu mol\;m^{-2}\;s^{-1}\right)$ affect plant photosynthetic rate and morphology, which is usually not incorporated in plant growth models. In this study, using data for indoor-grown lettuce (*Lactuca sativa*) cultivated under different light spectra, a mathematical model that incorporates these effects is presented. Different experimental cases are used to obtain a modified quantum use efficiency coefficient that varies with the spectral distribution. Several models for this coefficient are fitted using experimental data. Comparing the accuracy of these models, a simple first- or second-order linear model for light-use efficiency coefficient has about 6 to 8 percent uncertainty, while a fourth-order model has a 2 percent average error in prediction. In addition, normalizing overall spectral distribution leads to a more accurate prediction of the investigated parameter. A novel mathematical model based on normalized spectral irradiance integrated over wavelength for photosynthetically active radiation (PAR) wavebands and the far-red waveband is presented in this study. It accurately predicts lettuce dry mass grown indoors under different light spectra.

## 1 Introduction

The photon flux density and spectrum can independently and interactively affect crop photosynthesis, secondary metabolism, and other physiological processes (Ooms et al., 2016). Paz et al. (2019) investigated the impact of DLI (i.e., daily light integral), varying from 1.6 to 9.7 mol m^−2^ day^−1^ on the growth of indoor-grown red-leaf lettuce and suggested a minimum DLI of 6.5 mol m^−2^ day^−1^. However, biomass of lettuce continues to increase with DLI until some saturating value, when appearance of physiological disorders begin to appear (e.g., around 17 mol m^−2^ day^−1^) (Both et al., 1994; Kelly et al., 2020). While it is common for crops to have species- and cultivar-specific DLI recommendations for maximized growth rate, the spectral distribution at a constant DLI has additional impacts on biomass accumulation and morphology. For example, decreasing the red to far-red ratio (R:FR) typically increases extension growth (e.g., greater leaf area or elongated stems) that often increases per-plant biomass as a result of increased photon interception (Park and Runkle, 2018a; Park and Runkle, 2018b; Park and Runkle, 2019; He et al., 2021). Similarly, increasing the fraction of blue (B) light a plant receives inhibits extension growth and light interception and can decrease the per-plant biomass of lettuce (Meng et al., 2019; Park and Runkle, 2019; Kong and Nemali, 2021). Increasing the fraction of B and UV light can also increase the biosynthesis of secondary metabolites like anthocyanins which act as photo-protectants and can influence the photosynthetic rate (Meng and Runkle, 2019; He et al., 2021). As an additional consideration to the effect of light intensity and spectral distribution, PAR does not have a constant quantum yield of photosynthesis (mol CO2 assimilated per mol photon absorbed) on a per-nanometer basis; red light typically has a greater quantum yield than blue or green light (Hogewoning et al., 2012). Meng et al. (2020) analyzed the interaction of blue and green light on hydroponic lettuce growth, and replacing green with red light increased the quantum yield of photosynthesis.

Innovations have been made with respect to spectral-shifting materials for agricultural use that attempt to leverage our understanding of how light intensity and spectrum influence crops. For example, Shen et al. (2021) developed a spectral-shifting film that primarily absorbs blue and green light and fluoresces red and far-red light to theoretically increase lettuce biomass accumulation through increased quantum efficiency and light interception. Hebert et al. (2022) constructed luminescent quantum dot films that decrease overall DLI by 14%, but the modified spectrum enhances the tomato biomass yield and vegetative growth by 6% and 10%, respectively. Despite the wealth of knowledge on how light intensity and distribution affect crop growth, models predicting crop growth have not developed at a similar rate.

A plant growth model is a valuable tool to predict yield and provide an approximation for the impact of factors (e.g., water use or CO2 concentration). In addition, plant growth modeling allows researchers to perform virtual studies to test a hypothesis without investing the required time to perform costly experiments. Van Henten and Van Straten (1994) developed a dynamic model to predict lettuce dry mass as a state variable in time using environmental inputs such as CO_2_ concentration, spectral irradiance for photosynthetically active radiation, and ambient temperature. Jones et al. (1991) proposed a model for tomato growth that responds to constantly varying environmental parameters, and the plant state was presented through seven variables that included dry mass for different components, leaf number, and leaf area. These are two of several computational models that consider environmental parameters to increase crop yield. While these models consider the impacts of spectral distribution through the overall spectral irradiance (overall energy of the incoming spectrum), the impacts of spectral distribution on a photometric basis are often disregarded. There are few models in the literature that incorporate the impact of the photon spectrum of incoming light on plant growth. Dieleman et al. (2019) aimed to investigate the impact of light quality on tomato physiological and morphological responses. Young tomato plants were cultivated under 7 different light treatments, and various parameters were measured, including leaf light reflection and transmission, accumulated biomass, photosynthesis rate, and concentration of light-capturing pigments. Based on these measurements and the 3D model developed in GroIMP, and when extrapolated to a mature (fruit-bearing crop), it was suggested that dynamic light spectra might stimulate growth and production for an indoor crop production system.

The aim of this study is to modify an existing calibrated dynamic growth model of lettuce to accommodate the impact of spectral distribution. Several regression scenarios are investigated to find a modified model that estimates the impact of spectral distribution and intensity on plant growth. A new modified light-use efficiency coefficient that quantifies the impact of spectral distribution is also presented below.

## 2 Plant growth computational modeling

### 2.1 Plant growth model for lettuce

The dynamic growth model of lettuce proposed by Van Henten (1994) is modified in this study to numerically investigate the impact of spectral distribution and intensity on lettuce dry mass and yield. Using dry mass as the primary output for the model, this variable is further subdivided into structural dry mass and nonstructural dry mass, which accounts for starch, glucose, and other similar elements. The model assumes that the two categories of dry mass fully define the state of the plant and describes lettuce growth by calculating these sub-variables using the following ordinary differential equations (ODE),


(1) $$\frac{dX_{nsdm}}{dt}=c_{\alpha }f_{phot}-r_{gr}X_{sdm}-f_{resp}-\frac{1-c_{\beta }}{c_{\beta }}r_{gr}X_{sdm},$$

(2) $$\frac{dX_{sdm}}{dt}=r_{gr}X_{sdm}.$$

Equations (1) and (2) represent the transient behavior in the structural and non-structural dry mass per unit of area $\left(g\;m^{-2}\right)$ in response to photosynthesis. In the above equations, $f_{phot}=f_{phot}{\mathrm{(C}}_{CO_{2}},I,T,X_{sdm})$ is the gross canopy photosynthesis $\left(g\;m^{-2}\;s^{-1}\right)$ , $f_{resp}=f_{resp}\left(T,X_{nsdm},X_{sdm}\right)$ is the maintenance respiration $\left(g\;m^{-2}\;s^{-1}\right)$ , and $r_{gr}=r_{gr}\left(T,X_{nsdm},X_{sdm}\right)$ is the growth rate of structural material $\left(g\;m^{-2}\;s^{-1}\right)$ , while $c_{\alpha }$ and $c_{\beta }$ describe the conversion rate of CO $2_{\;}$ to sugar (CH ${\;}_{2}$ O) and yield factor which is a measure of non-structural dry mass losses due to respiration and photosynthetic activities, respectively. The value for $c_{\alpha }$ is the molecular weight ratio of CO ${\;}_{2}$ to CH ${\;}_{2}$ O and is set to 0.68 . According to Sweeney (1981), $c_{\beta }$ for lettuce is approximately 0.8 . The growth rate $\left(r_{gr}\right)$ refers to the rate at which non-structural materials are transformed into structural materials, i.e,


(3) $$r_{gr}=c_{gr,max}\frac{X_{nsdm}}{c_{\gamma }X_{sdm}+X_{nsdm}}c_{Q_{10},gr}^{\left(T-20\right)/10},$$

where T is the canopy temperature (°C) , $c_{gr,max}$ is the saturation growth rate at 20°C , $c_{\gamma }$ is the growth rate coefficient, and $c_{Q_{10},gr}$ is the measure of growth rate sensitivity to the canopy temperature. Van Holsteijn (1981) approximated the saturation growth rate coefficient to $5\times 10^{-6}\;s^{-1}$ ; Sweeney (1981) estimated the growth rate coefficient for lettuce to 1.0 . The growth rate sensitivity constant is set to 1.6 , which means that for every 10°C increase in the canopy temperature, the growth rate increases by a factor of 1.6 . The maintenance respiration rate is predicted through,


(4) $$f_{resp}=\left(c_{resp,sht}\left(1-c_{\tau }\right)X_{sdm}+c_{resp,rt}c_{\tau }X_{sdm}\right)c_{Q_{10},resp}^{\left(T-25\right)/10}.$$

In Equation (4), $c_{resp,sht}$ and $c_{resp,rt}$ represent shoot and root maintenance respiration coefficients at 25°C and indicate the amount of glucose consumption per structural dry material. Van Henten (1994) estimated shoot and root respiration coefficients as $3.47\times 10^{-7}\;s^{-1}$ and $1.16\times 10^{-7}\;s^{-1}$ , respectively. $c_{Q_{10},resp}$ is the sensitivity of maintenance respiration to canopy temperature and Van Henten (1994) assigned a value of 2.0 for this coefficient. $c_{\tau }$ is the ratio of root dry mass to the overall dry mass of the plant, which can depend on the type of cultivation. Lorenz and Wiebe (1980)reported an average value of 0.15 for lettuce cultivated in soil, while Sakamoto and Suzuki (2015) measured an average value of 0.14 for hydroponic lettuce cultivation. Goudriaan and Monteith (1990) formulated an empirical correlation to estimate gross canopy photosynthesis,


(5) $$f_{phot}=\left(1-\exp\;\left(-c_{K}c_{lar}\left(1-c_{\tau }\right)X_{sdm}\right)\right)f_{phot,max},$$

that $c_{K}$ is the extinction coefficient, and for lettuce with planophile characteristics, is set to 0.9 ; $c_{lar}$ is the structural leaf area ratio and Lorenz and Wiebe (1980) approximated it to $75\times 10^{-3}\;m^{2}\;g^{-1}$ ; and $f_{phot,max}$ is the gross CO ${\;}_{2}$ assimilation rate for a canopy with 1 square meter of effective surface area. Acock et al. (1978) presented an equation to calculate $f_{phot,max}$ considering the effect of CO ${\;}_{2}$ concentration and spectral irradiance integrated over wavebands within PAR as well as canopy temperature and photorespiration,


(6) $$f_{phot,max}=\frac{\varepsilon Ig_{CO_{2}}c_{\omega }\left(C_{CO_{2}}-\Gamma \right)}{\varepsilon I+g_{CO_{2}}c_{\omega }\left(C_{CO_{2}}-\Gamma \right)}.$$

In Equation (6), ε is the light-use efficiency, I is the spectral irradiance integrated over the wavebands within PAR that regulates plant growth, $g_{CO_{2}}$ is the conductance of canopy for the diffusion of CO ${\;}_{2}$ , $c_{\omega }$ is the density of CO ${\;}_{2}$ that has an approximate value of $1.83\times 10^{-3}\;g\;m^{-3}$ (considering greenhouse temperature around 20 ${\;}^{\circ }$ C), $C_{CO_{2}}$ is the concentration of CO ${\;}_{2}$ in the greenhouse, and Γ is the CO ${\;}_{2}$ compensation point, accounting for the impact of the temperature on photosynthesis rate (Van Henten, 1994). CO ${\;}_{2}$ compensation is determined based on canopy temperature using the following correlation,


(7) $$\text{Γ}=c_{\text{Γ}}c_{Q_{10},\text{Γ}}^{\;\;\;\;\;\;\;\left(T-20\right)/10},$$

whereas $c_{\text{Γ}}$ is the CO ${\;}_{2}$ compensation point at 20°C which is $40\;mL\;L^{-1}$ , and $c_{Q_{10},\text{Γ}}$ is the sensitivity of CO ${\;}_{2}$ compensation with canopy temperature, which Goudriaan et al. (1985) approximated it as 2.0 . Light-use efficiency is computed considering light level impact on CO ${\;}_{2}$ compensation and photorespiration (Goudriaan et al., 1985),


(8) $$\varepsilon =c_{\varepsilon }\frac{C_{CO_{2}}-\Gamma }{C_{CO_{2}}+2\Gamma }.$$

In Equation (8), $c_{\varepsilon }$ is the quantum use efficiency which is the energy required for a reduction of one mole CO ${\;}_{2}$ , and Goudriaan et al. (1985) approximated its value to be about $17.0\times 10^{-6}\;g\;J^{-1}$ . In this study, there is an assumption that this parameter is affected by the photon spectral distribution; therefore, its value varies depending on the lighting conditions utilized for lettuce growth. Goudriaan et al. (1985) developed a mathematical correlation for the canopy conductance for CO ${\;}_{2}$ diffusion, which is derived considering the boundary layer, stomatal, and carboxylation conductance,


(9) $$\frac{1}{g_{CO_{2}}}=\frac{1}{g_{bnd}}+\frac{1}{g_{stm}}+\frac{1}{g_{car}},$$

where $g_{bnd}$ , $g_{stm}$ , and $g_{car}$ represent the boundary layer, stomatal, and carboxylation conductance, respectively. Stanghellini (1987) estimated the boundary layer conductance to be $0.007\;m\;s^{-1}$ at a 5°C temperature gradient, 0.1 $m\;s^{-1}$ wind speed, and leaf with a characteristic length of 0.075 m . For a plant that grows in an environment without stress, Stanghellini (1987) approximated stomatal conductance to be $0.005\;m\;s^{-1}$ . Carboxylation conductance is a function of canopy temperature and its value (from 5 to 40°C) is determined using the following empirical correlation,


(10) $$g_{car}=-1.32\times 10^{-5}T^{2}+5.94\times 10^{-4}T-2.64\times 10^{-3}.$$

**Table 1** provides a summary for the definition of different coefficients and their numerical values within the plant growth model.

**Table 1:** Summary of coefficients needed in Equations (1)-(9) for lettuce cultivation modeling.

Parameter	Definition	Value	Reference
$c_{\alpha }$	Conversion rate of CO_2_ to CH_2_O	0.68	Van Henten (1994)
$c_{\beta }$	Yield factor	0.8	Sweeney (1981)
$c_{gr,max}$	Saturation growth rate at 20°C	$5\times 10^{-6}\;s^{-1}$	Van Holsteijn (1981)
$c_{\gamma }$	Growth rate coefficient	1.0	Sweeney (1981)
$c_{Q_{10},gr}$	Growth rate sensitivity to the canopy temperature	1.6	Sweeney (1981)
$c_{resp,sht}$	Shoot maintenance respiration coefficient at 25°C	$3.47\times 10^{-7}\;s^{-1}$	Van Henten (1994)
$c_{resp,rt}$	Root maintenance respiration coefficient at 25°C	$1.16\times 10^{-7}\;s^{-1}$	Van Henten (1994)
$c_{Q_{10},resp}$	Sensitivity of maintenance respiration to the canopy temperature	2.0	Van Henten (1994)
$c_{\tau }$	Ratio of root dry mass to total plant dry mass (soil)	0.15	Lorenz and Wiebe (1980)
	Ratio of root dry mass to total plant dry mass (hydroponic)	0.14	Sakamoto and Suzuki (2015)
$c_{K}$	Extinction coefficient	0.9	Goudriaan and Monteith (1990)
$c_{lar}$	Structural leaf area ratio	$75\times 10^{-3}\;m^{2}\;g^{-1}$	Lorenz and Wiebe (1980)
$c_{\omega }$	Density of CO ${\;}_{2}$	$1.83\times 10^{-3}\;g\;m^{-3}$	Van Henten (1994)
$c_{\text{Γ}}$	CO ${\;}_{2}$ compensation point at 20°C	$40\;mL\;L^{-1}$	Goudriaan et al. (1985)
$c_{Q_{10},\text{Γ}}$	Sensitivity of CO_2_ compensation with canopy temperature	2.0	Goudriaan et al. (1985)
$c_{\varepsilon }$	Quantum use efficiency as energy required for a reduction of one molecule of CO_2_	$17.0\times 10^{-6}\;g\;J^{-1}$	Goudriaan et al. (1985)
$g_{bnd}$	Boundary layer conductance	$0.007\;m\;s^{-1}$	Stanghellini (1987)
$g_{stm}$	Stomatal conductance	$0.005\;m\;s^{-1}$	Stanghellini (1987)

### 2.2 Plant growth ODE solver

**Figure 1:**
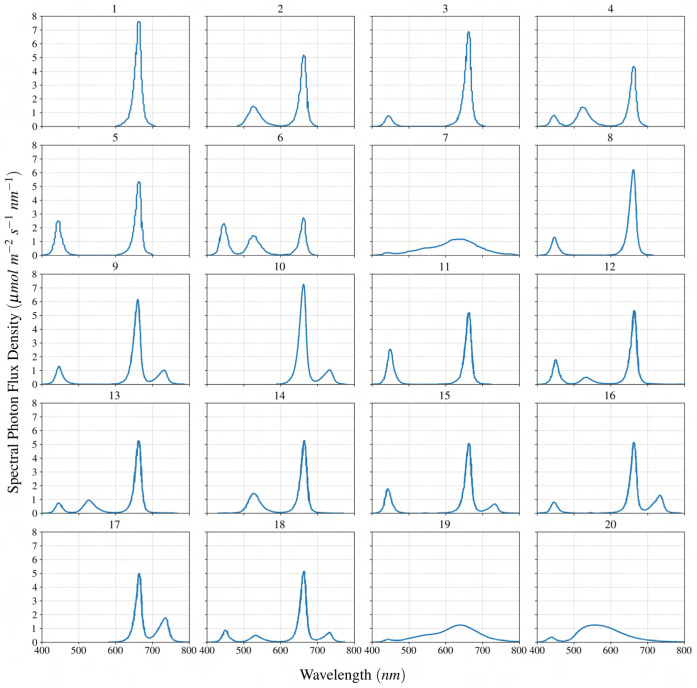
Spectral distribution for different case studies of lighting treatment for lettuce reported in the literature to study the effect of spectral distribution on lettuce growth. The label at the top of each graph represents a light treatment experiment according to **Table 2**.

A MATLAB code was developed to find a solution for the ODE Equations (1), and (2). The code utilized experimental temperature, spectral irradiance integrated over wavebands from 400 to 750 nm , and CO ${\;}_{2}$ concentration as inputs to compute the two sub-variable dry masses as outputs. Input and output data were extracted from experiments that investigated the impact of the photon spectrum on production of lettuce ‘Rouxai’ growth by Meng and Runkle (2019); Meng et al. (2019), and Meng et al. (2020). Meng and Runkle (2019) carried out three replications with a PPFD of 100 and 180 $\mu mol\;m^{-2}\;s^{-1}$ during 0-2 and 2-3 days, respectively. After that, the seedlings were grown under various LED treatments with a 24-hour photoperiod, and the temperature was set to 23 ° C. Fresh and dry mass data were obtained using destructive tests for plants harvested on day 10. Similarly, Meng et al. (2019) performed experiments three times at a total photon flux density of 180 $\mu mol\;m^{-2}\;s^{-1}$ . The seedlings were transplanted into a hydroponic system with a 20-hour photoperiod, an average air temperature of 21.1 ${\;}^{\circ }$ C, average CO ${\;}_{2}$ concentration of 402 $mL\;L^{-1}$ , and relative humidity ranging from 41 % to 70%. Meng et al. (2020) conducted two replications at a temperature of 20 ° C, a total photon flux density of 50 $\mu mol\;m^{-2}\;s^{-1}$ , and 24-hour photoperiod. The next day, the temperature, the photoperiod, and total photon flux density were set to 22 ° C, 20 hours, and 180 $\mu mol\;m^{-2}\;s^{-1}$ , respectively. On the fourth day, the seedlings were exposed to nine different light-quality treatments under the same controlled conditions. For the first replication, the average temperature, relative humidity, and CO ${\;}_{2}$ concentration was 22.4 °C, 410 $mL\;L^{-1}$ , and 34% , and in the second replication, these parameters were 22.4 °C, 410 *mL L*-1 and 35%, respectively. **Table 2** represents the experimental data for red-leaf lettuce ‘Rouxai’ under different light treatment conditions, while **Figure 1** illustrate the variation of incoming light spectra.

**Table 2:** Experimental data for different spectral treatments obtained from Meng and Runkle (2019) and Meng et al. (2019, 2020).

TreatmentNumber	Literature	TreatmentType	Dry Mass(g)	PFD $\left(\mu mol\;m^{-2}s^{-1}\right)$	I PAR+FR $\left(Wm^{-2}\right)$
1	Meng et al. (2020)	R180	2.931	180.2	32.8
2	Meng et al. (2020)	G60R120	2.745	184.3	36.3
3	Meng et al. (2020)	B20R160	2.258	179.6	34.4
4	Meng et al. (2020)	B20G60R100	2.449	180.2	37.2
5	Meng et al. (2020)	B60R120(1)	2.051	183.0	38.5
6	Meng et al. (2020)	B60G60R60	1.527	178.7	40.3
7	Meng et al. (2020)	WW180(1)	2.470	184.8	36.5
8	Meng and Runkle (2019)	B30R150	0.046	183.7	36.2
9	Meng and Runkle (2019)	B30R150FR30	0.052	216.5	41.7
10	Meng and Runkle (2019)	R180FR30	0.045	214.2	38.4
11	Meng et al. (2019)	B60R120(2)	1.014	178.1	37.3>
12	Meng et al. (2019)	B40G20R120	1.187	182.5	37.3
13	Meng et al. (2019)	B20G40R120	1.348	181.7	36.4
14	Meng et al. (2019)	G60R120	1.587	184.3	36.3
15	Meng et al. (2019)	B40R120FR20	1.232	180.8	36.1
16	Meng et al. (2019)	B20R120FR40	1.438	183.6	34.4
17	Meng et al. (2019)	R120FR60	1.622	174.2	30.1
18	Meng et al. (2019)	B20G20R120FR20	1.417	182.4	35.4
19	Meng et al. (2019)	WW180(2)	1.394	188.1	37.0
20	Meng et al. (2019)	EQW180	1.087	181.7	38.4

*B, G, R, FR, WW*, and *EQW* refers to blue (400–500nm), green (500–600nm), red (600–700nm), far-red (700–750nm), warm-white, and equalized white light-emitting diodes, respectively according to Meng et al. (2020). *PFD*, and *I_PAR+FR_* represent photon flux density and spectral irradiance integrated over PAR and far-red wavebands.

**Figure 2:**
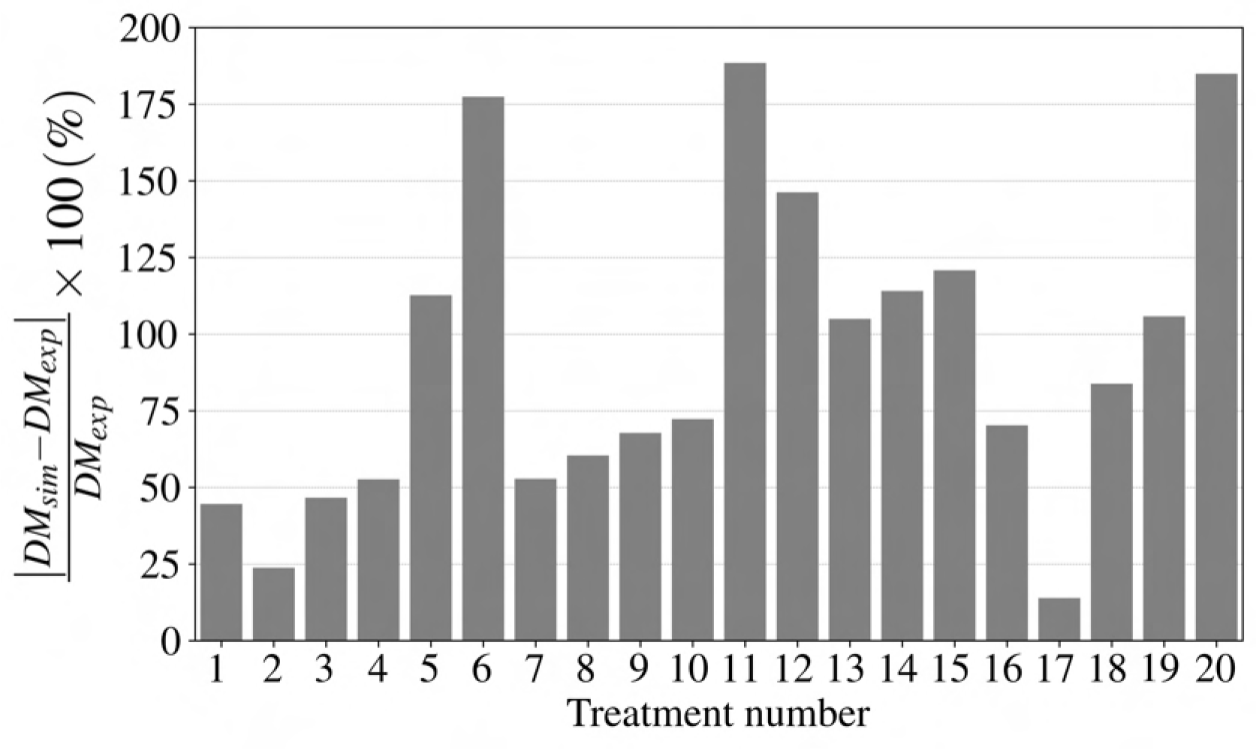
Numerical error for the lettuce growth model using the $c_{\varepsilon }$ value by Van Henten (1994). *DM_sim_*, and *DM_exp_* are lettuce dry mass for numerical simulation and experimental study in *g*, respectively. The considerabledifferences, some with more than 75 % error, indicate the necessity of considering the impact of spectral irradiance and flux density on the $c_{\varepsilon }$ value for an accurate prediction of lettuce growth yield.

Using integrated spectral irradiance, CO ${\;}_{2}$ concentration, and temperature, the Van Henten (1994) growth model is used with the parameters in **Table 1** to predict dry mass for different light treatment experiments. **Figure 2** represents the necessity of considering the impact of spectral distribution on lettuce growth by showing the difference between the experimental data and the plant growth model. For example, the growth model predicted only 6 of the 20 lighting treatments to be within 25 % of the actual dry mass values.

Numerical error is the measure of a difference between the experimental dry mass of lettuce and the model prediction using the suggested value for $c_{\varepsilon }$ (Van Henten, 1994), which was $17.0\times 10^{-6}\;g\;J^{-1}$ . With the assumption of unvarying $c_{\varepsilon }$ for different experiments, the dynamic growth model does not accurately predict for various light treatment experiments other than typical greenhouse light conditions.

### 2.3 Validation of ODEs solver through altering $c_{\varepsilon }$ for different experiments

**Figure 3:**
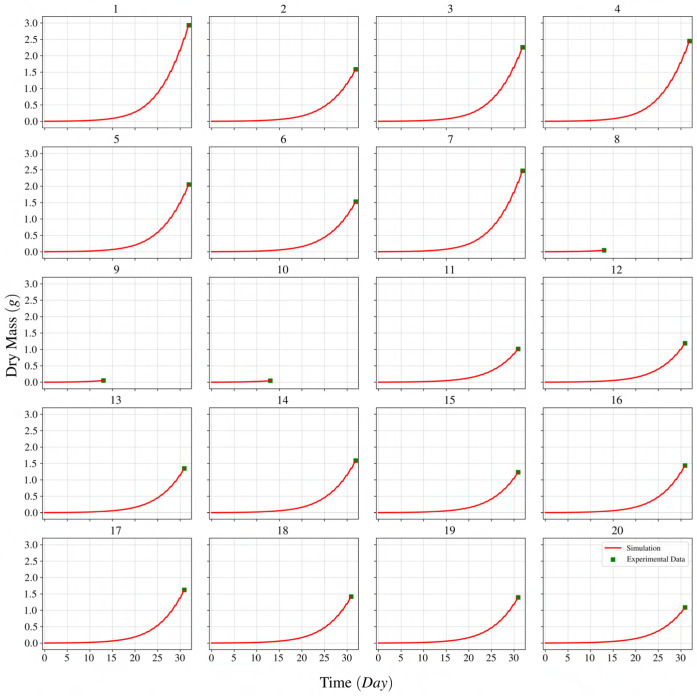
Comparison of lettuce dry mass for a dynamic growth model with experimental data under different spectral distributions and intensities. The label at the top of each graph represents a light treatment experiment according to **Table 2**.

As mentioned earlier, using a constant $c_{\varepsilon }$ led to a considerable error in lettuce dry mass prediction; however, as we will show, a varying $c_{\varepsilon }$ depending on spectral distribution allows prediction in good accordance with experimental data. Knowing the dry mass of lettuce for different experiments, the solver tries to find a value for $c_{\varepsilon }$ that allows a prediction of a state variable in good accordance with experimental data. These values will be used in the next section to develop a model that predicts the impact of the photon spectrum on $c_{\epsilon }$ , and eventually on plant growth. **Figure 3** compares the lettuce dry mass predicted by the model with the experimental dry mass for the investigated light treatments. It is inferred from **Figure 3** that the solver is capable of finding a quantum use efficiency for each experiment that leads to an accurate prediction of the lettuce state variable (dry mass) on the day of harvest.

## 3 Implementation of regression methodology to account for the impact of spectral distribution and intensity on lettuce growth

This section describes the development of the linear regression model. Two distinctive datasets are considered, which represent the experiments carried out under different LED spectrums and novel data for natural light. The general form of the model is presented in the next subsection, which is followed by a discussion on the input features of the model. Exploratory data analysis is performed on the input dataset in subsection 3.3. The generic form of the empirical models is investigated in subsection 3.4, and the performance of different models is evaluated using various metrics such as *R* ^2^, mean absolute percentage error (MAPE), Akaike information criterion (AIC), and Bayesian information criterion (BIC), in the next subsection. A regression model is built from the LED lighting data using a train and test split of 85% and 15%, respectively. The effects of combining the suggested empirical model with the dynamic growth model for lettuce are studied in subsection 3.6. In the last subsection, the precision of the proposed combined dynamic growth model is evaluated using data from another study conducted under completely different experimental conditions under natural lighting.

### 3.1 Light-use efficiency prediction based on incoming spectrum

**Figure 4:**
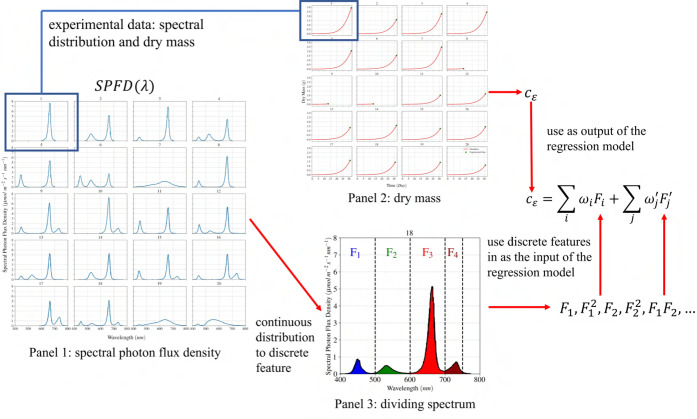
The development of the regression model for light-use efficiency based on spectral photon flux density distribution is shown. In Panel 1 (from **Figure 1**), λ is the wavelength (nm), and SPFD is the spectral photon flux density ( μ mol m ${\;}^{-2}$ s ${\;}^{-1}$ nm ${\;}^{-1}$ ). The continuous SPFD is converted to four discrete features, as seen in Panel 3 (from **Figure 5**). Dry mass versus time is given in Panel 2 (from **Figure 3**), which is then converted to c ${\;}_{\varepsilon }$ . $c_{\varepsilon }$ , given on the right, is the light-use efficiency in the dynamic growth model of lettuce, and F ${\;}_{i}$ and $\omega_{i}$ are the discrete input features based on the incoming spectrum and the corresponding weights, respectively. Finally, Terms with prime correspond to the interaction between different wavebands.

The aim of this study is to develop a model that predicts the quantum use efficiency $\left(c_{\varepsilon }\right)$ as a function of spectral photon flux density or integrated spectral irradiance within the PAR+FR waveband (i.e., 400-750 nm). A linear regression approach proves to be an invaluable tool for generating a basic model for obtaining weights for different features and establishing a simple mathematical model in the form of $c_{\epsilon }=\sum_{i=1}^{4}\left(\omega_{i}F_{i}\right)$ where $\omega_{i}$ and $F_{i}$ correspond to the weight (coefficient) and the value for the i ${\;}^{\text{th}}$ input feature, respectively. Since the incoming spectrum is a continuous function (**Figure 1**), an idea was devised to generate discrete features based on the continuous distribution of the spectrum for the linear regression model, as shown in **Figure 4**. This idea involves dividing the incoming spectrum into four segments, which in the context of this study, corresponding to four distinctive wavebands in PAR+FR: blue (400-500 nm), green (500-600 nm), red (600-700 nm), and far-red (700-750 nm). A discrete value is assigned for each segment based on the integral of spectral distribution. Subsection 3.2 provides a detailed description of how these discrete values are obtained for each spectrum.

In addition, it is possible to investigate the interaction between different light wavebands on quantum use efficiency through the regression model in the form of ${\bar{c}}_{\epsilon }=\sum_{i=1}^{4}\left(\omega_{i}F_{i}\right)+\sum_{j}\left(\omega_{j}^{{\;}^{'}}F_{j}^{{\;}^{'}}\right)$ where $F_{j}^{{\;}^{'}}$ can be defined as the multiplication of two fraction ratios, e.g., the blue and green wavebands. **Figure 4** provides an overview of the development of empirical correlation for light-use efficiency based on the continuous spectrum distribution.

### 3.2 Definition of the fraction ratio

**Figure 5:**
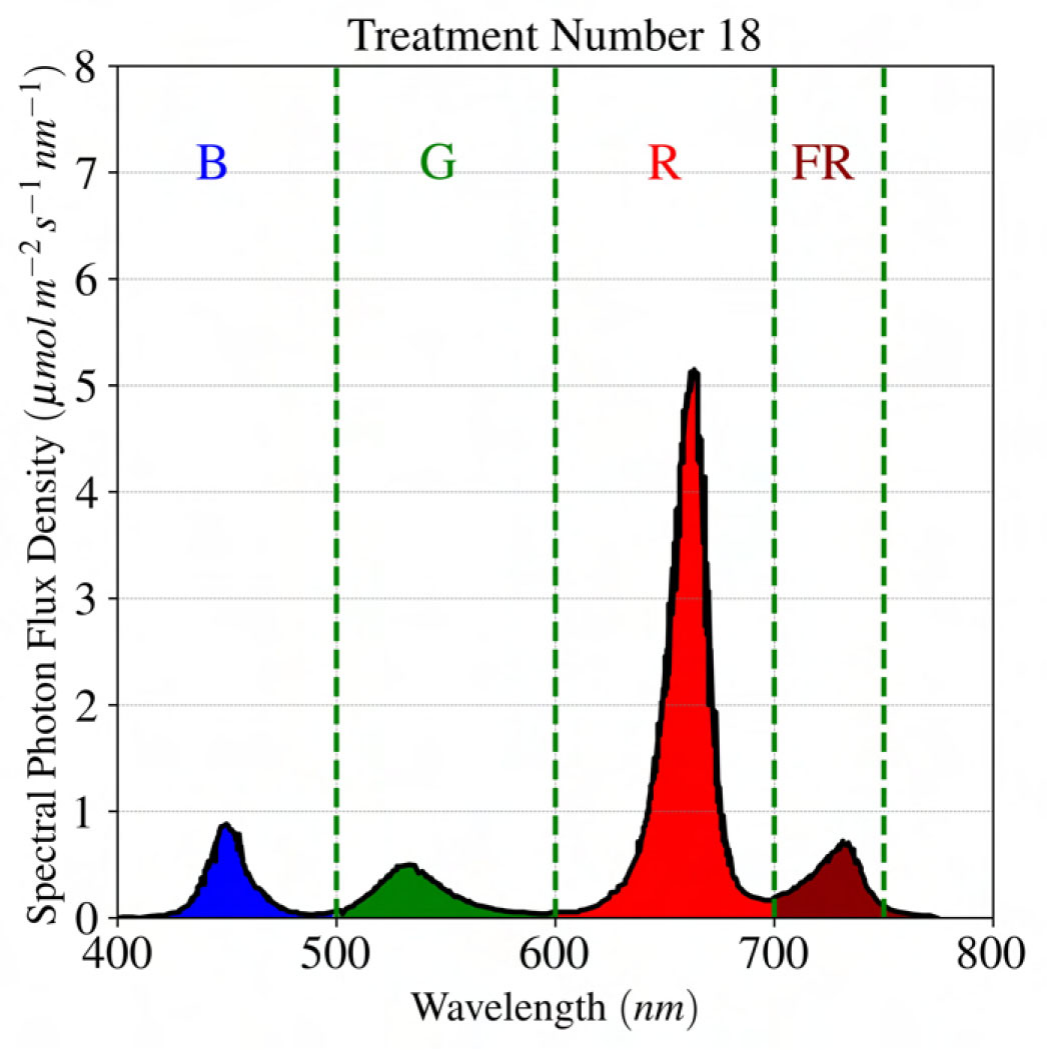
Dividing the photon spectrum for experimental treatment number 18, or “B20G20R20FR120”, to calculate the intensity ratio that corresponds with the integrated spectral irradiance or PFD distribution. B, G, R, FR corresponds to blue (400−500 nm) , green (500−600 nm) , red (600−700 nm) , and far-red (700−750 nm) wavebands.

As previously stated, the dynamic growth model’s efficiency can be improved by developing a model for $c_{\varepsilon }$ that takes into account the impact of spectral distribution. This can be accomplished by creating a function for $c_{\varepsilon }$ that is dependent on the photon flux density or integrated spectral irradiance ratio for 100-nm wavebands in PAR and 50-nm FR waveband. Calculation of these ratios based on spectral photon flux distribution is more convenient since Meng et al. (2020) assigned a label based on photon flux density treatments with different wavebands. Considering lighting treatment 18, or “B20G20R120FR20” as an example, the photon flux density for blue and green light is $20\;\mu mol\;m^{-2}\;s^{-1}$ , red light is $120\;\mu mol\;m^{-2}\;s^{-1}$ , and far-red light is $20\;\mu mol\;m^{-2}\;s^{-1}$ . The photon flux density for different wavebands represents the areas under the curve in **Figure 5**. Therefore, photon flux density ratios for the blue, green, and far-red wavebands are $\frac{20}{180}$ , while for the red waveband is equal to $\frac{120}{180}$ . Computation of the ratios for integrated spectral irradiation of the PAR+FR wavebands is different, since spectral irradiance is a measure of the energy carried by a photon. Therefore, integrated spectral irradiance (I) for a specific spectrum of PAR+FR is determined by calculating the energy for each wavelength through multiplication of wavelength energy and its number of photons and integrating those over the specific waveband. For “B20G20R120FR20” as an example, the integrated spectral irradiance for blue, green, red and far-red wavebands are $5.57\;W\;m^{-2}$ , $4.57\;W\;m^{-2}$ , $21.74\;W\;m^{-2}$ , and $3.51\;W\;m^{-2}$ , respectively. Based on the integrated spectral irradiance values computed for different wavebands, the integrated spectral irradiance intensity ratios for blue, green, red, and far-red are $\frac{5.57}{35.38}$ , $\frac{4.57}{35.38}$ , $\frac{21.74}{35.38}$ , and $\frac{3.50}{35.38}$ , or 15.7:12.9:61.4:9.9 respectively.

### 3.3 Exploratory data analysis

**Figure 6:**
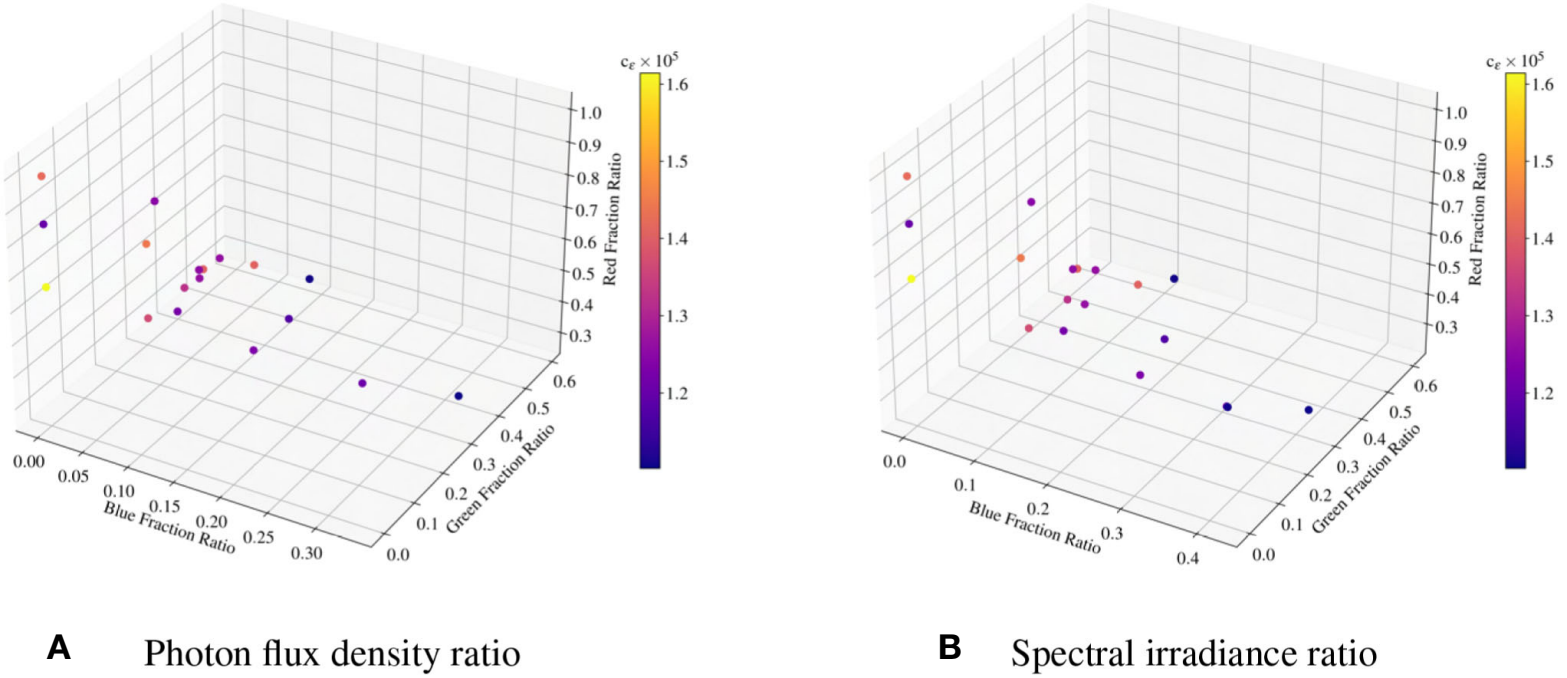
3D visualizations of fraction ratio distribution for photon flux density and spectral irradiance ratio. The color bar represents the value of the light-use efficiency for various experiments.

With the calculation of $c_{\varepsilon }$ from the experimental data, the next step is to create regression models to fit polynomial functions over a set of discrete variables and predict the light-use efficiency. As the aim of this study is to establish a framework that can estimate light-use efficiency as a function of incoming spectra, the input features include discrete parameters associated with either PFD or spectral irradiance distribution. The first set of input variables consists of photon flux density ratios for blue, green, red, and far-red wavebands, which are calculated by integrating the photon flux density distribution shown in **Figure 1**. On the other hand, the second set of input parameters includes spectral irradiance ratios for the same wavebands, obtained by integrating over spectral irradiance distribution for various lighting distributions. Before investigating various regression models, the properties of the data used for regression are explored. **Table 3** represents the mean, standard deviation (std), minimum (min), maximum (max), and percentile values for the investigated features (25% or first quartile, 50% or second quartile or median, 75% or third quartile), the input (F is the fraction ratio which is either based on photon flux density (PFD) or spectral irradiance integrated over wavelengths (I) whereas B, G, R, and FR represent blue, green, red, and far-red wavebands) and the output (c ${\;}_{\varepsilon }$ is the light-use efficiency) of the model. **Figure 6** visualizes the 3D distribution for photon flux density and spectral irradiance fraction ratios for the studied dataset.

**Table 3:** Statistical information for ratios based on integrated spectral and photon flux density distributions, and $c_{\varepsilon }$ .

Variable	Mean	Std	Min	25%	50%	75%	Max
*F_B,PFD_*	0.127	0.112	0	0.049	0.111	0.181	0.333
*F_G,PFD_*	0.147	0.178	0	0	0.056	0.309	0.588
*F_R,PFD_*	0.660	0.167	0.284	0.639	0.667	0.679	1
*F_FR,PFD_*	0.066	0.093	0	0	0	0.111	0.333
*F_B,I_*	0.168	0.142	0	0.065	0.155	0.250	0.421
*F_G,I_*	0.158	0.187	0	0	0.063	0.322	0.605
*F_R,I_*	0.614	0.174	0.254	0.562	0.612	0.665	1
*F_FR,I_*	0.060	0.088	0	0	0	0.097	0.321
$c_{\varepsilon }\times 10^{5}$	1.30	0.142	0.110	1.21	1.26	1.40	1.61

Mean, std, min, and max represent the average, standard variation, minimum, and maximum values in the dataset. 25 % , 50 % , and 75 % represent numerical values for the first quartile, median, and third quartile (based on the assumption that data is sorted in ascending order). F is the fraction ratio which is either based on photon flux density (PFD) or spectral irradiance integrated over wavelengths (I), whereas B, G, R, and FR represent blue, green, red, and far-red wavebands, and c ${\;}_{\varepsilon }$ is the light-use efficiency.

### 3.4 Predictive model including polynomial features for the quantum use efficiency coefficient ( $c_{\varepsilon }$ )

Different polynomial models examined within the aim of this study have a form similar to


(11) $$c_{\epsilon }=a_{0}+\sum_{i=1}^{4}\left[a_{i}F_{i}+b_{i}F_{i}^{2}+c_{i}F_{i}^{3}+d_{i}F_{i}^{4}+\sum_{j=i}^{4}\left(e_{ij}F_{i}F_{j}\right)\right].$$

Not all of the models have every term presented in Equation (11), e.g., the regression model based on the first-order term is defined as ${\bar{c}}_{\varepsilon }=a_{0}+\sum_{i=1}^{4}(a_{i}F_{i})$ . Regression models are defined in a way that includes up to 16 weight coefficients. Within the scope of this study, 22 distinctive terms are investigated, that are provided in Equation (11), and includes ratios ( $F_{i}$ , 4 terms representing each waveband), the square of ratios ( $F_{i}^{2}$ , 4 terms), the cubic of ratios ( $F_{i}^{3}$ , 4 terms), the quartic of ratios ( $F_{i}^{4}$ , 4 terms), and interaction ratios ( $F_{i}F_{j}$ , 6 terms). Therefore, studied regression models within the scope of this study are comprised of linear models with 4 (includes $F_{i}$ terms), 8, 12, 14, and 16 (includes $F_{i}$ , $F_{i}^{2}$ , $F_{i}^{3}$ , $F_{i}^{4}$ terms) weight coefficients. Initially, a regression model with four terms corresponding to the first-order terms was developed and investigated as a baseline model. Higher-order polynomials were then explored to improve the poor performance (R ${\;}^{2}$ < 0.45%) of this linear model. Polynomials with 8 (combinations of linear, second-order, and interaction terms), 12 (same with third-order terms), 14 (same as 12 with two more terms), and 16 (including fourth-order terms) were considered. It should be noted that all 22 terms (all combinations through fourth-order) were not used because of the small size of the dataset. Results from these model choices will be given in the following section.

**Figure 7:**
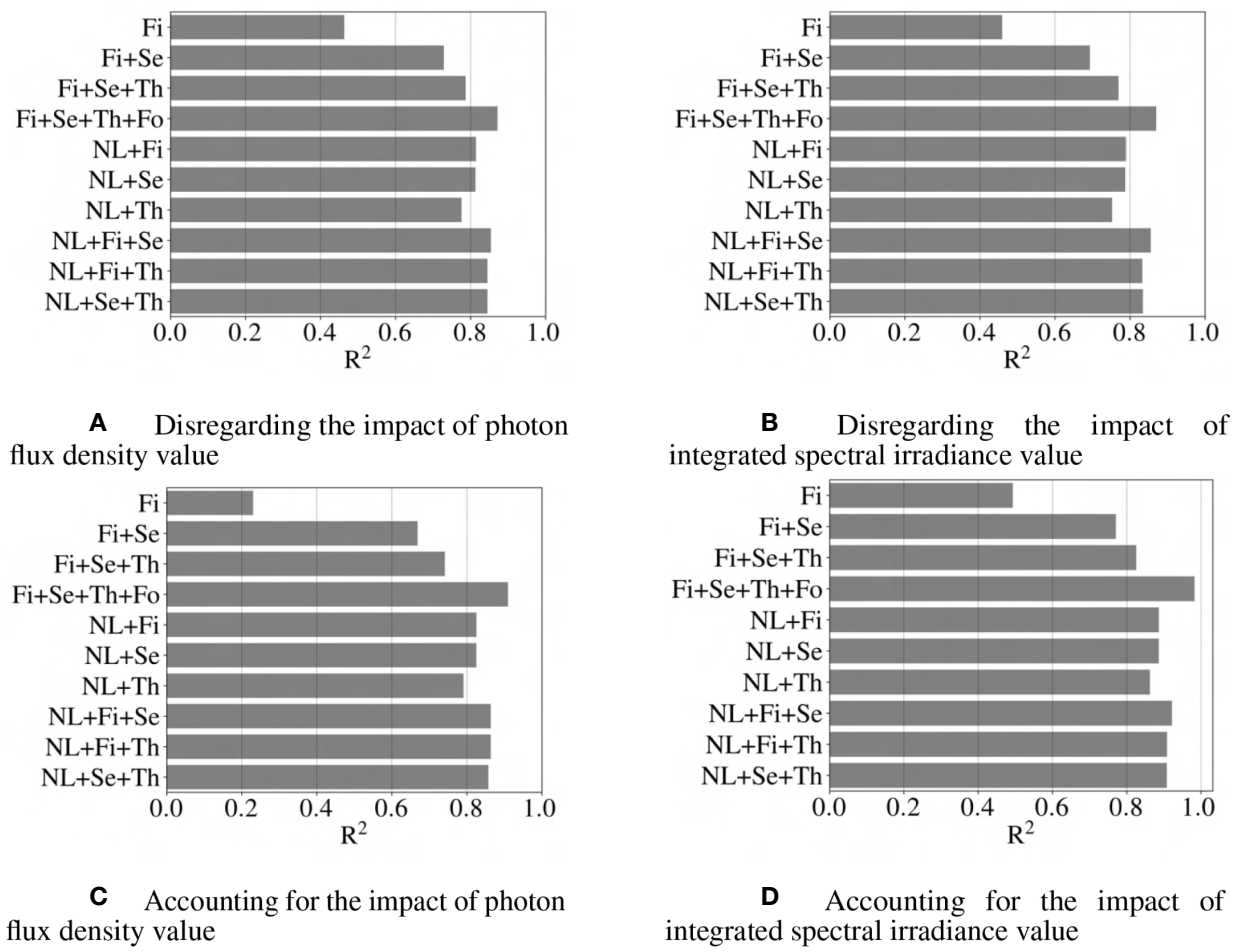
Comparison of different models based on R^2^ score criteria. Abbreviations within these figures are NL, nonlinear (includes 6 interaction terms between wavebands), Fi, first-order terms; Se, second-order terms; Th, third-order terms; Fo, fourth-order terms. R^2^ score closer to 1 indicates a more accurate model.

Since the dataset is comprised of 20 observations of $c_{\varepsilon }$ for different spectral distributions and intensities (**Figure 1**), increasing the number of coefficients beyond the suggested limit would result in an overfitted model. In other words, this would lead to a model capable of accurate prediction for the studied data; however, evaluating the performance of the model against new data would decrease the fidelity of the model. The term “nonlinear” in this section refers to polynomial models based on Equation (11) in which $e_{ij}\neq 0$ for every i and j value. The regression models which estimate the impact of incoming light spectrum on the light-use efficiency are classified into the following categories: 1) models based on PFD ratios that disregard the impact of overall photon flux density; 2) models based onintegrated spectral irradiance ratios that disregard the impact of overall value; 3) models based on PFD ratios, that considered the impact of overall photon flux density; 4) models based on integrated spectral irradiance ratios that considered the impact of overall value. **Figures 7A–D**, represent the accuracy of categories 1, 2, 3, and 4 using R ${\;}^{2}$ metric.

To prevent overfitting and ensure the model’s applicability to new data, a validation study is conducted on the dataset. This involves reserving a portion of the data for testing, which is not used during the model training phase. The testing data is used to evaluate the model’s performance on new and unseen data. The goal is to find a model that performs well on both training data and testing data, thereby preventing underfitting or overfitting issues. For instance, for a regression model with 16 weight coefficients, 3 samples are randomly chosen for testing purposes. The remaining 17 samples are used to train the regression model, and then its accuracy is evaluated on the 3 unseen samples. The selected regression model (and the corresponding weights for different terms) is the one that performs well on both training data (17 samples) and testing data (3 samples). This approach ensures that the model is not overfitting and can predict well on new data, making it useful for practical applications. In addition to using unseen data for the validation of the regression model, a regularization penalty (L1 or L2 norm) is introduced into the regression model to decrease the variation caused by the complexity of the model.

### 3.5 Development and performance comparison of regression models

**Figure 8:**
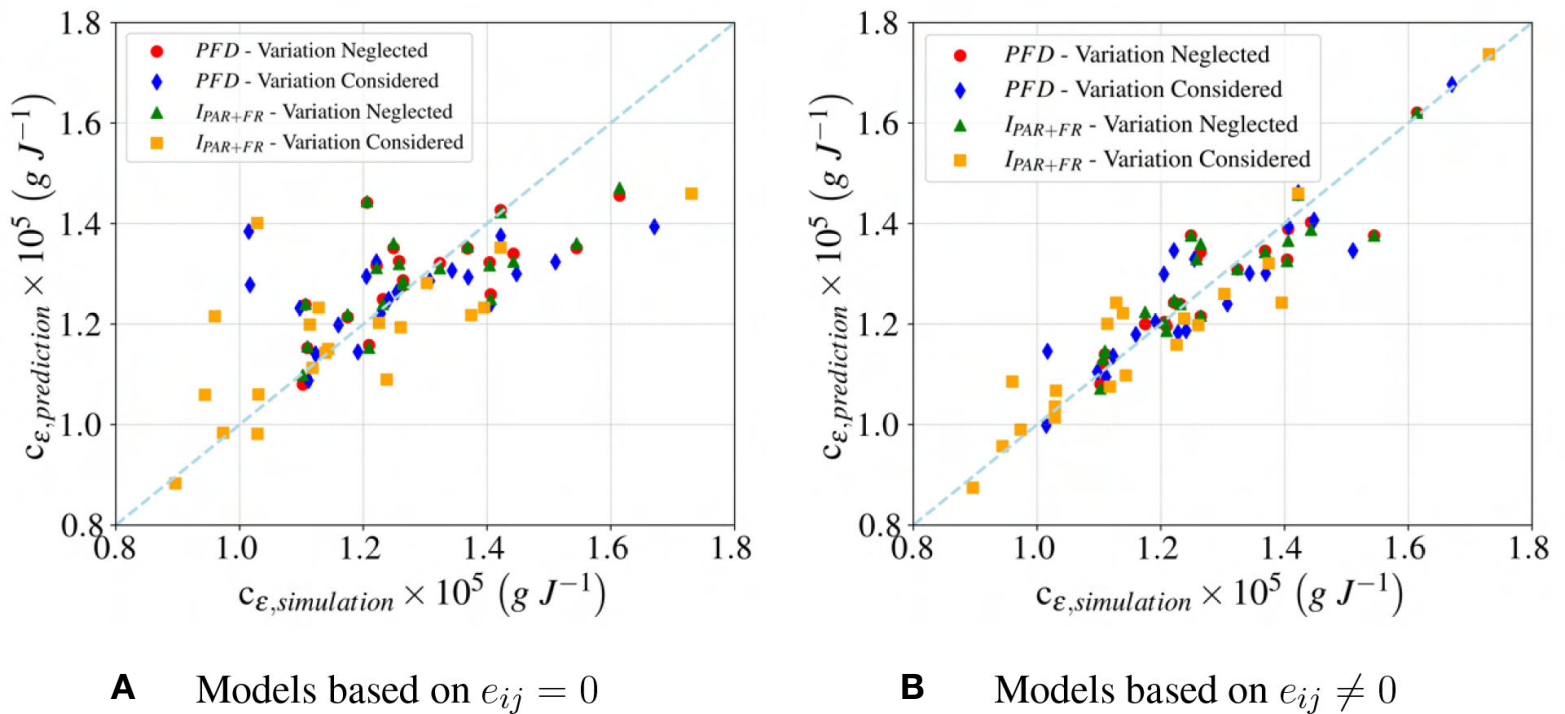
Accuracy of first-order regression models with the assumption of **(A)** Neglecting nonlinearity (eij = 0), and **(B)** Considering nonlinearity (eij /= 0). IPAR+FR refers to a regression model based on theintegrated spectral irradiance ratio, while PFD represents a model based on the photon flux density ratio.

**Figure 9:**
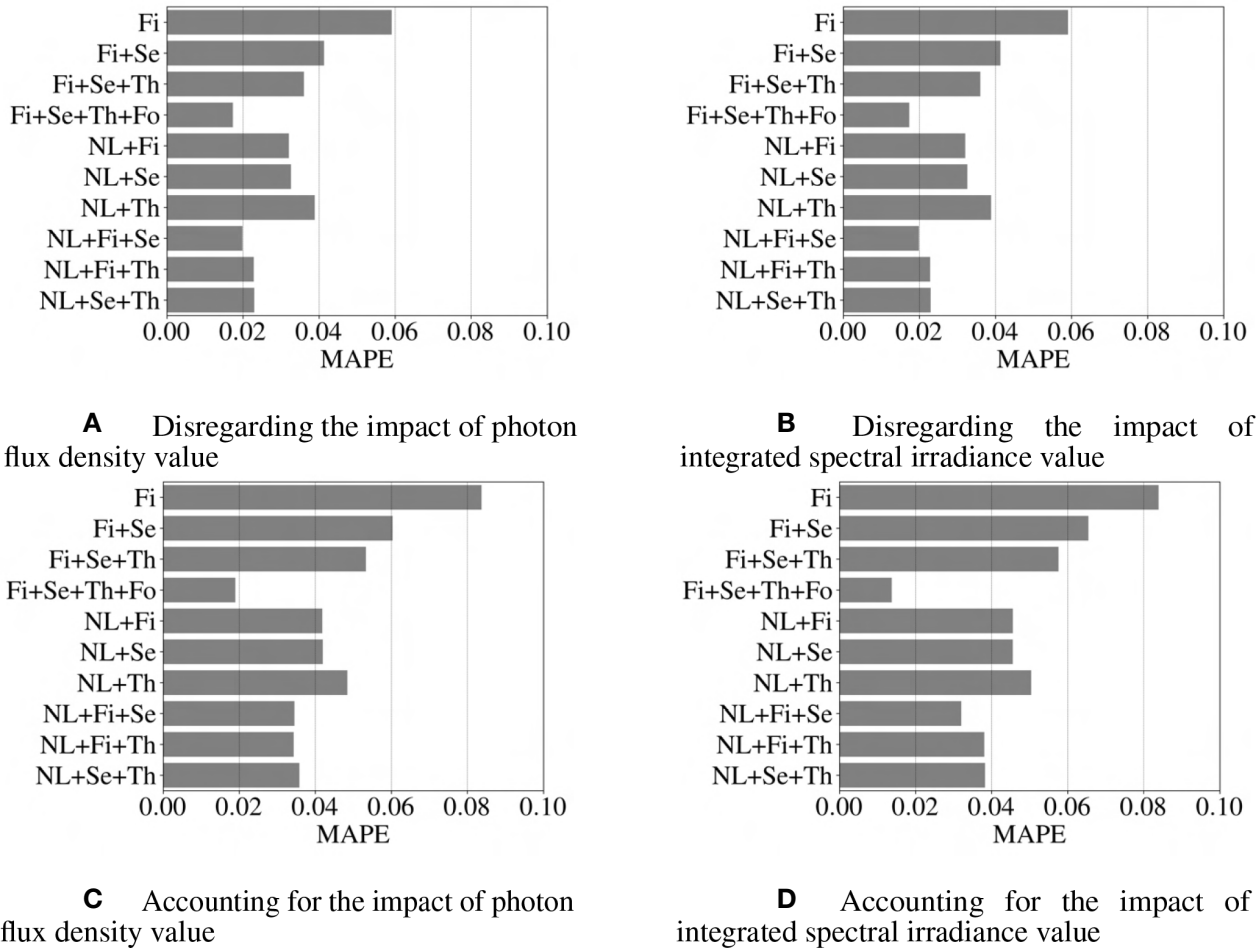
Comparison of different models based on the mean absolute percentage error (MAPE) score criteria. Abbreviations within these figures are NL, nonlinear (includes 6 interaction terms between wavebands), Fi, first-order terms; Se, second-order terms; Th, third-order terms; Fo, fourth-order terms. The lower value for MAPE score indicates a more accurate model.

**Figure 10:**
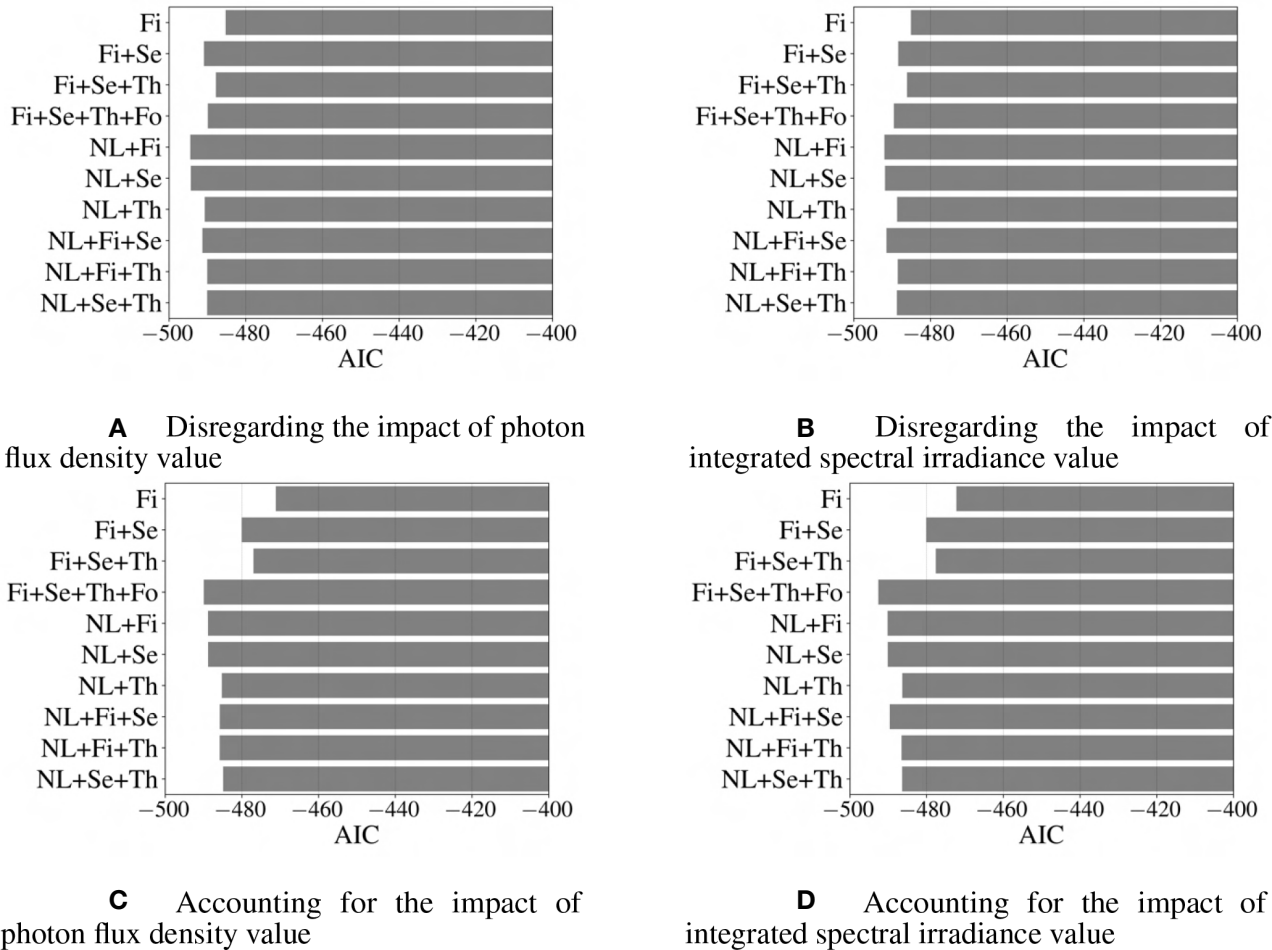
Comparison of different models based on AIC criteria. Abbreviations within these figures are NL, nonlinear (includes 6 interaction terms between wavebands), Fi, first-order terms; Se, second-order terms; Th, third-order terms; Fo, fourth-order terms. The lower the value for AIC, the better the fit of the model (Baguley, 2018).

**Figure 11:**
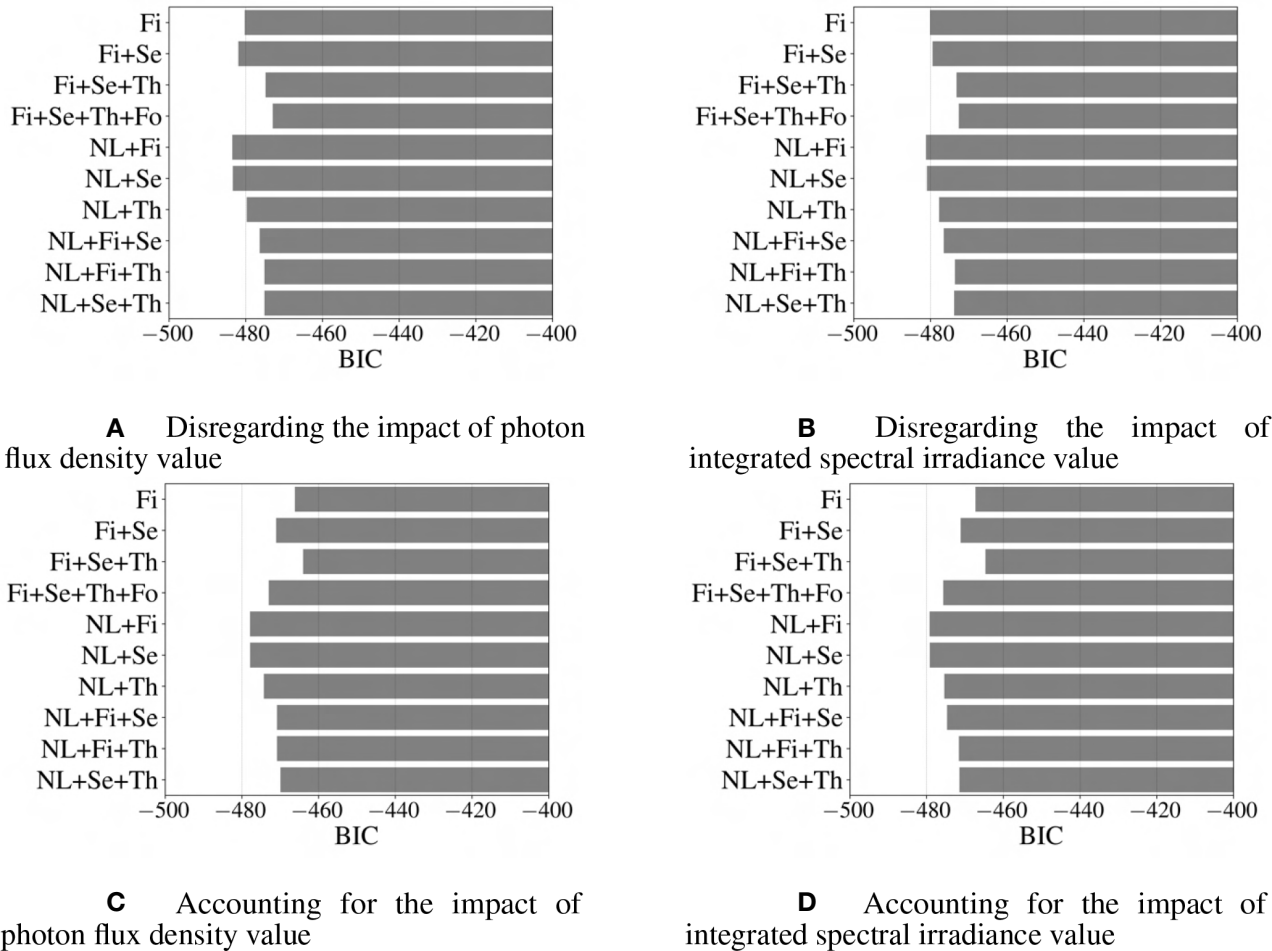
Comparison of different models based on BIC criteria. Abbreviations within these figures are NL, nonlinear (includes 6 interaction terms between wavebands), Fi, first-order terms; Se, second-order terms; Th, third-order terms; Fo, fourth-order terms. The lower the value for BIC, the better the fit of the model (Baguley, 2018).

In this study, the Scikit-learn built-in function *LinearRegression* developed by Pedregosa et al. (2011) is used to build a model that minimizes the regularized residual sum of squares (R ${\;}^{2}$ ) as the criteria for the closest linear functionto the actual data ^1^. The R ${\;}^{2}$ score is calculated using R ${\;}^{2}=1-\frac{\sum_{i=1}^{n}{\left(y_{i}-{\hat{y}}_{i}\right)}^{2}}{\sum_{i=1}^{n}{\left(y_{i}-y\right)}^{2}}$ , in which $y_{i}$ and ${\hat{y}}_{i}$ represented the true and predicted value for the i ${\;}^{th}$ sample and y is the average of the actual values. In addition to $R^{2}$ criteria, the mean absolute percentage error (MAPE) is also calculated for different models and is defined as MAPE $=\frac{1}{n_{samples}}\sum_{i=1}^{n_{samples}}\frac{\left|y_{i}-{\hat{y}}_{i}\right|}{max\;(\epsilon ,\left|y_{i}|\right)}$ , where ϵ is an arbitrary non-zero small positive number to ensure that MAPE is defined. **Figure 8** compares the actual value, c ${\;}_{\varepsilon ,simulation}$ , versus the predicted value, c ${\;}_{\varepsilon ,prediction}$ : a straight line would indicate a perfect prediction. In **Figure 8A**, only terms linear in the features $F_{i}$ are included; in contrast, in **Figure 8B**, first-order and interaction terms in Equation (11) are used. It is seen that the accuracy increases with more features. **Figure 8** provide qualitative insight into the accuracy, whereas R ${\;}^{2}$ and MAPE provide quantitative metrics, as shown in **Figures 7**, **9**. According to **Figures 7**, **9**, in spite of simplicity for the first-order and the combination of first and second-order models, these models have poor accuracy in predicting the $c_{\varepsilon }$ as a function of spectral distribution with R ${\;}^{2}$ score less than 0.75 . This is addressed by considering several models created from the general expression in Equation (11): ten different combinations of features are shown, each as a horizontal bar. Not all combinations are shown in the **Figures 7**, **9**–**11**; a representative set for a different number of terms is visualized, but one that includes the best performing model (16 terms).

Normalization of c ${\;}_{\varepsilon }$ to accommodate the impact of overall integrated spectral irradiance or photon flux density was examined and found to increase the fidelity of the regression model. Note the improvement for the particular case of a model that includes first-, second-, third-, and fourth-order terms (Fi+Se+Th+Fo bar in **Figure 7**), which we will establish as the best model below. For this case, using normalization resulted in a 16-feature regression model that has an average error of 2%, as compared to the un-normalized error of 15%.

In addition to R ${\;}^{2}$ and MAPE metrics, the Akaike information criterion (AIC) and the Bayesian information criterion (BIC) are also computed for the studied models, which are the measures of the model’s complexity. The value of AIC and BIC for models with a constant parameter is obtained using the following correlations (Seabold and Perktold, 2010): AIC = −2×LL+log (N)×(k+1) , and BIC = −2×LL+2×(k+1) , whereas LL is the log of the likelihood function (how likely it is that the model predicted the actual values), N is the size of training data, and k is the number of features, respectively. It is worth mentioning that a lower value of AIC or BIC indicates that the fitted model is a better fit for the data, as it balances the goodness of fit and the complexity of the model model (Baguley, 2018). **Figures 10**, **11** provide visual representations of the impact of the model’s complexity on AIC and BIC value. For the studied dataset, by increasing the accuracy of the model through the addition of new terms, the likelihood function also improves, which ultimately outweighs the negative penalty associated with the higher number of features (k); therefore, for the investigated models, the introduction of new features into the regression model would generally decrease AIC value. **Figure 11** demonstrates that the BIC shows a different pattern, where regression models including nonlinear terms and only one of the first-, second-, or third-order terms are better fitted than those consisting ofnonlinear terms and two of the first-, second-, or third-order terms.

It is important to note that while a lower AIC or BIC value suggests a better model, it does not necessarily mean that the model is the best possible fit for the data. In order to determine the best-fitted model, a comprehensive analysis of the performance of the studied models is conducted based on AIC, BIC, MAPE, and R ${\;}^{2}$ metrics. Based on this comparison, it is evident that the model, which incorporates first-, second-, third-, and fourth-order terms based on the integrated spectral irradiance ratio considering the impact of overall integrated spectral irradiance of light-use efficiency, performs better than the other models with higher accuracy, and relatively lower AIC and BIC values.

**Figure 12:**
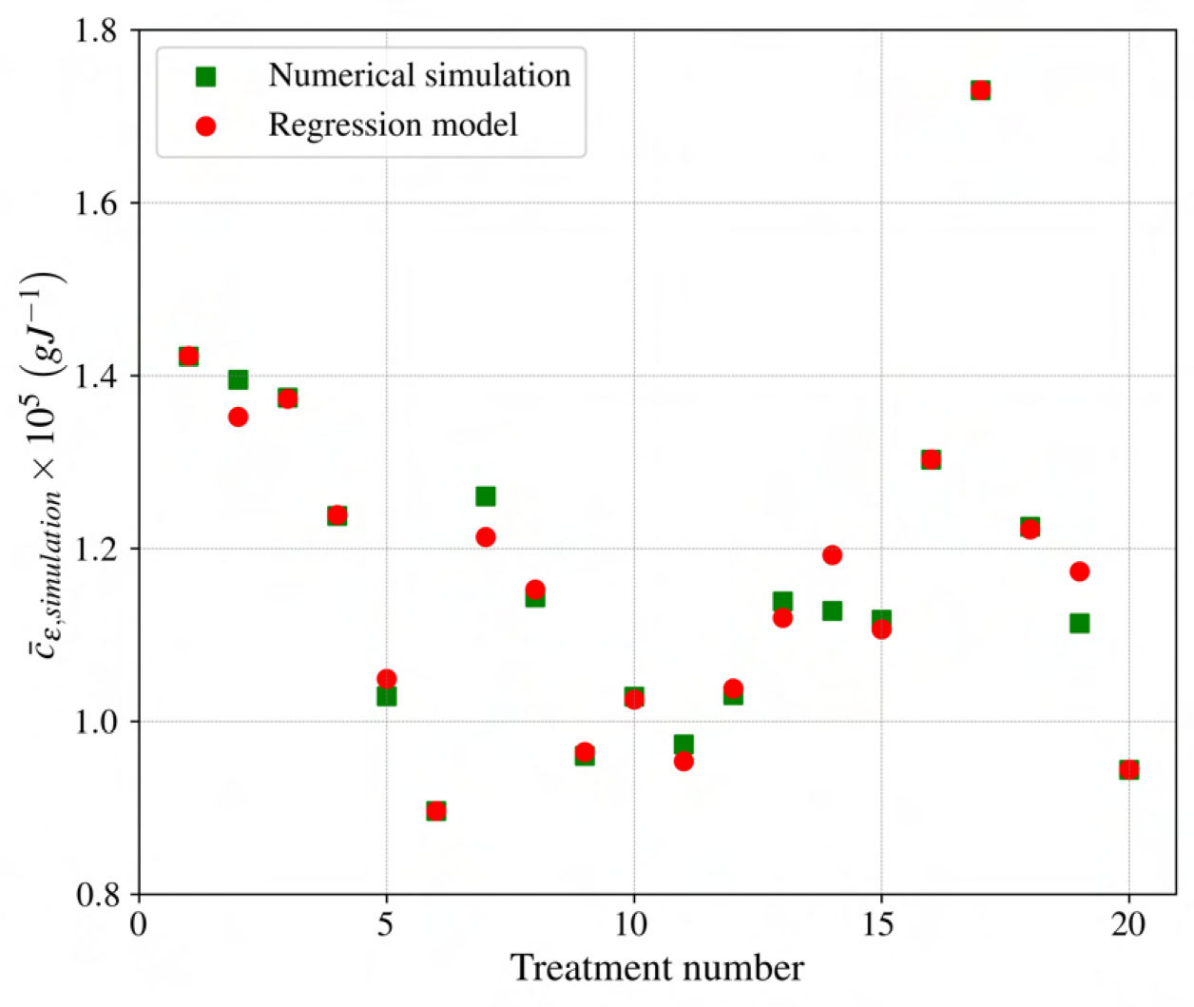
Comparison of $c_{\varepsilon }$ for the suggested model with numerical simulation for different light treatments.


Equation (12) demonstrates the high fidelity regression model based on spectral irradiance distribution normalized with integrated spectral irradiance for R180 light treatment based on **Table 2**. As shown in **Figure 12**, predictions of the suggested model are in good accordance with numerical data (for the best performing model, the testing data include treatment numbers 7, 14, and 19.); however, additional data could prove useful in developing a more comprehensive model.

(12) $$\begin{array}{l} c_{\epsilon }/R_{I}=-1.40\times 10^{-4}+1.82\times 10^{-4}F_{B,I}+2.06\times 10^{-4}F_{B,I}^{2}\\ \;\;\;-7.71\times 10^{-4}F_{B,I}^{3}+8.38\times 10^{-4}F_{B,I}^{4}+2.32\times 10^{-4}F_{G,I}\\ \;\;\;-4.20\times 10^{-4}F_{G,I}^{2}+1.53\times 10^{-3}F_{G,I}^{3}-1.56\times 10^{-3}F_{G,I}^{4}\\ \;\;\;-1.90\times 10^{-4}F_{R,I}+1.03\times 10^{-3}F_{R,I}^{2}-1.10\times 10^{-3}F_{R,I}^{3}\\ \;\;\;+4.13\times 10^{-4}F_{R,I}^{4}+4.68\times 10^{-4}F_{FR,I}-5.25\times 10^{-3}F_{FR,I}^{2}\\ \;\;\;+2.92\times 10^{-2}F_{FR,I}^{3}-4.77\times 10^{-2}F_{FR,I}^{4} \end{array}$$

Equation (12) is constrained by the following condition,


(13) $$F_{B,I}+F_{G,I}+F_{R,I}+F_{FR,I}=1$$

Now that the best-performing model is selected, the impact of adding higher-order terms to the regression model is investigated. The following notation $\left[a_{1}\;,\;a_{2}\;,\;a_{3}\;,\;a_{4}\right]$ is used to evaluate the impact of higher-order term addition on first-order terms, in which $a_{1}$ , $a_{2}$ , $a_{3}$ , and $a_{4}$ represent weights for blue, green, red, far-red fraction ratios,respectively. The examined models included a first-order term model with weights of $\left[-1.24\times 10^{-5}\;,\;-6.96\times 10^{-6}\;,\;-3.60\times 10^{-6}\;,\;-2.18\times 10^{-7}\right]$ , a combination of first- and second-order terms model with $\left[6.95\times 10^{-5}\;,\;7.34\times 10^{-5}\;,\;8.02\times 10^{-5}\;,\;4.29\times 10^{-5}\right]$ , a combination of first- to third-order terms model with $\left[6.89\times 10^{-6}\;,\;-2.41\times 10^{-5}\;,\;4.60\times 10^{-5}\;,\;-1.60\times 10^{-5}\right]$ , and a combination of first- to fourth-order terms model with $\left[1.82\times 10^{-4}\;,\;2.32\times 10^{-4}\;,\;-1.90\times 10^{-4}\;,\;4.68\times 10^{-4}\right]$ . Comparison between these notations suggests that the addition of higher-order terms to the regression models significantly affects the weights of the first-order terms, highlighting the importance of exploring more complex models with higher-order terms for improved prediction accuracy.

### 3.6 Coupling the regression model with van henten dynamic growth model for lettuce

**Figure 13:**
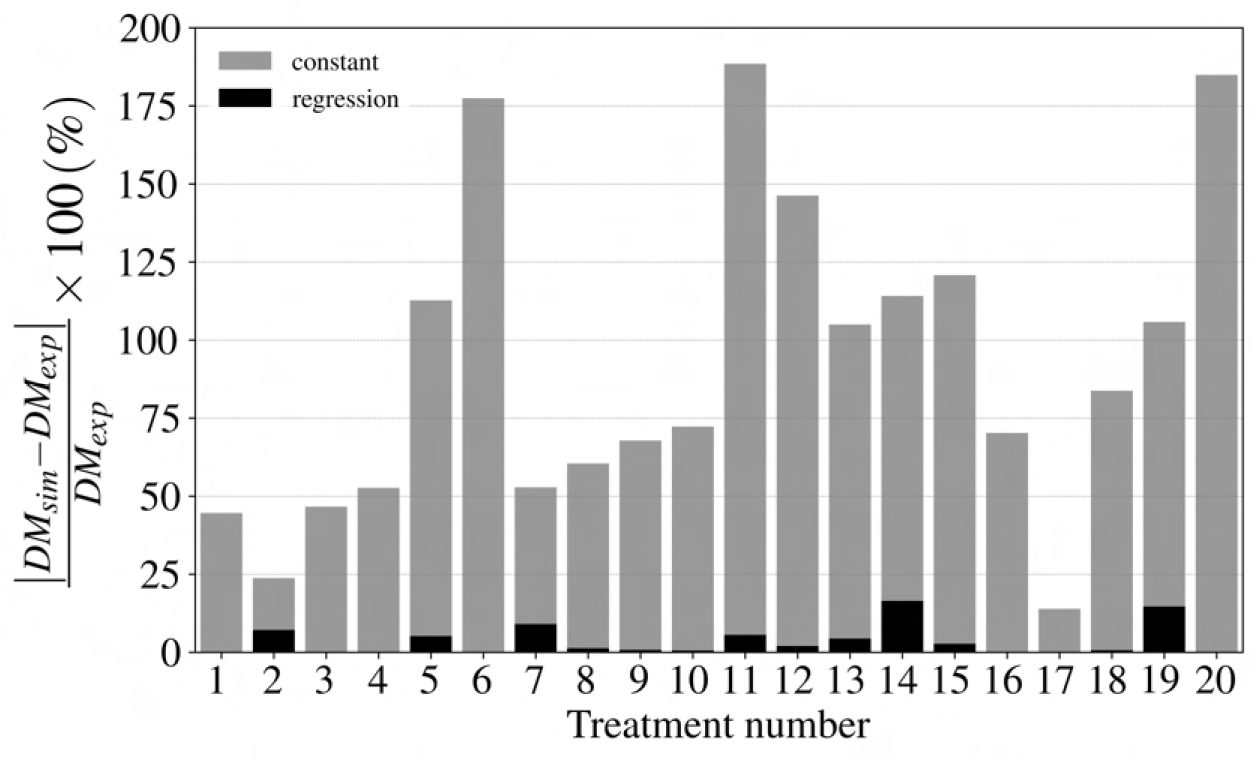
Computed numerical error is significantly reduced based on $c_{\varepsilon }$ value using Equation (12) (regression label, black bar) compared with error using the suggested constant value for $v_{\varepsilon }$ by Van Henten (1994) (constant label, light gray bar). $DM_{sim}$ , and $DM_{exp}$ are lettuce dry mass for numerical simulation and experimental study in g , respectively. As shown in the figure, coupling the regression model with the dynamic growth model improved the accuracy of prediction for lettuce cultivated under different spectral distributions.

Using Equation (12) and integrated spectral irradiance fraction ratio for the investigated wavebands of the different light treatment cases, the Van Henten (1994) dynamic growth model is utilized to compare the accuracy of the modified growth model that accounts for spectral distribution and intensity. **Figure 13** compares the difference between experimental dry mass for lettuce with dry mass prediction of the modified growth model.

Comparing the numerical error of the lettuce growth model using the suggested value for light-use efficiency (labeled constant in **Figure 13**), with that of the lettuce growth model withthe proposed regression model (Equation (11); labeled regression in **Figure 13**), it is inferred that the suggested regression modelof the $c_{\varepsilon }$ improves the accuracy of Van Henten (1994) growth model and adequately considers the impact of spectral distribution on plant growth.

### 3.7 Cross-validation of the proposed light-use efficiency model with novel data

**Figure 14:**
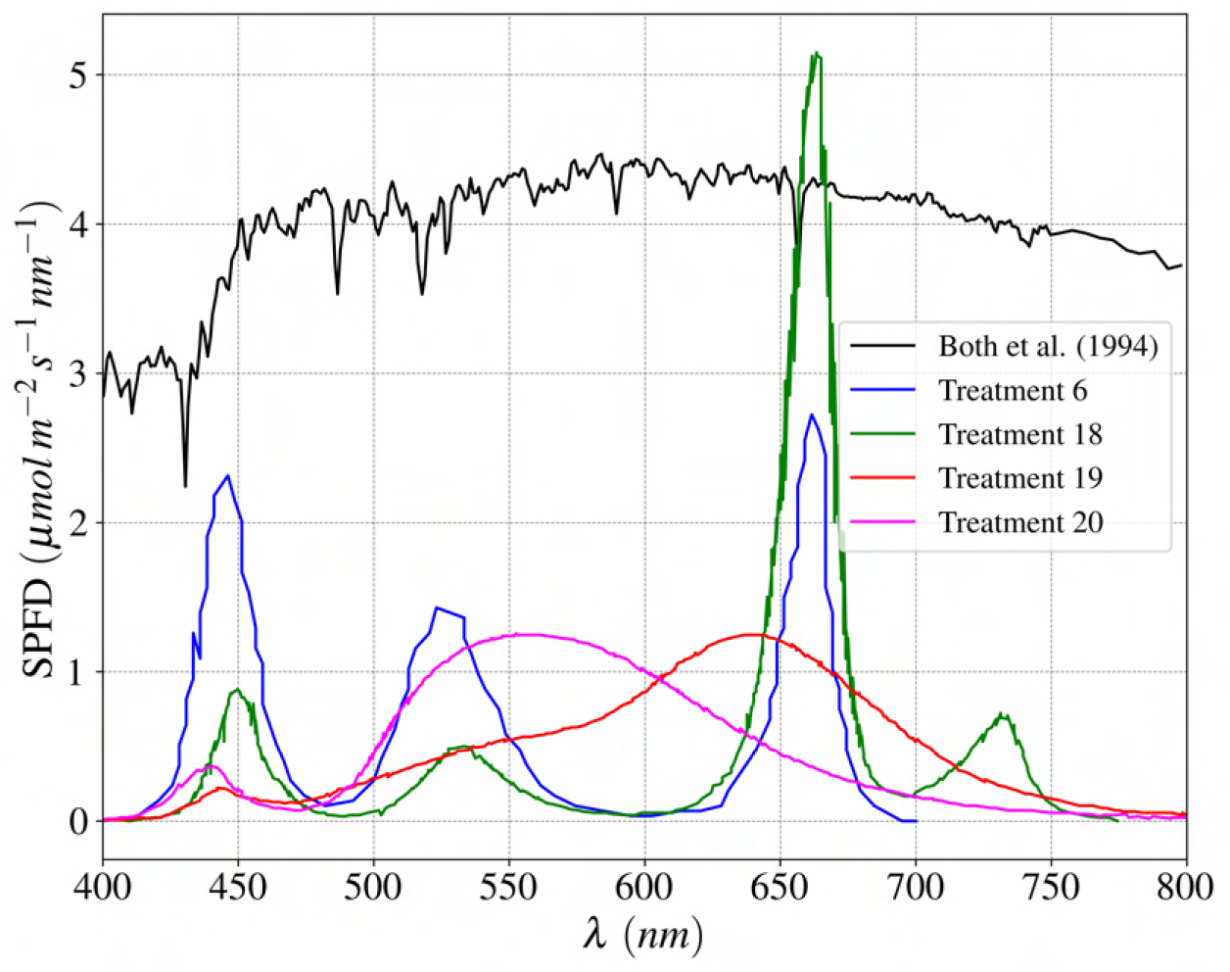
Comparison of spectral photon flux density distribution for natural light (Both et al., 1994) and investigated LEDs spectrum.

In the previous section, the performance of the proposed mathematical model is investigated through integration with the Van Henten (1994) dynamic growth model. To further investigate the accuracy of the light-use efficiency model based on the spectral irradiance ratios of various wavebands, data from Both et al. (1994) on lettuce cultivated in a controlled greenhouse environment is utilized. From October 1992 to March 1993, six controlled light treatments (without the use of supplemental lighting) were conducted for lettuce grown hydroponically. For these treatments, during the first 11 days, the temperature and CO ${\;}_{2}$ concentration were maintained at 25 °C and 350 mLL ^-1^, respectively. After day 11, the temperature was set to 24 °C between 7 am and 5 pm and 18.8 °C for the rest of the day, while CO ${\;}_{2}$ was enriched to 1000 *mL L^-1^*. **Table 4** represents data used for cross-validation of the suggested regression-based light-use efficiency model. Spectral irradiance intensity and distribution ratios are approximated using reported daily light integral for the different treatments and predicted solar irradiation by Tobiska et al. (2000). **Figure 14** displays a comparison of the spectral photon flux density distribution with respect to wavelength for the investigated LED spectrums and natural lighting.

**Table 4:** Experimental dry mass data for greenhouse cultivated hydroponic lettuce grown under controlled light treatments obtained by Both et al. (1994).

Experimental period	Daily light integral (mol m^–2^ day^–1^)	Dry Mass						
		Day 14	Day 18	Day 21	Day 25	Day 28	Day 32	Day 35
November 1992	6.2	0.064	0.153	0.3	0.76	1.01	1.735	2.46
January 1993	4.7	0.063	0.141	0.2	0.44	0.84	1.2	1.87
February 1993	10.5	0.085	0.288	0.53	1.21	1.98	3.15	4.81

**Figure 15:**
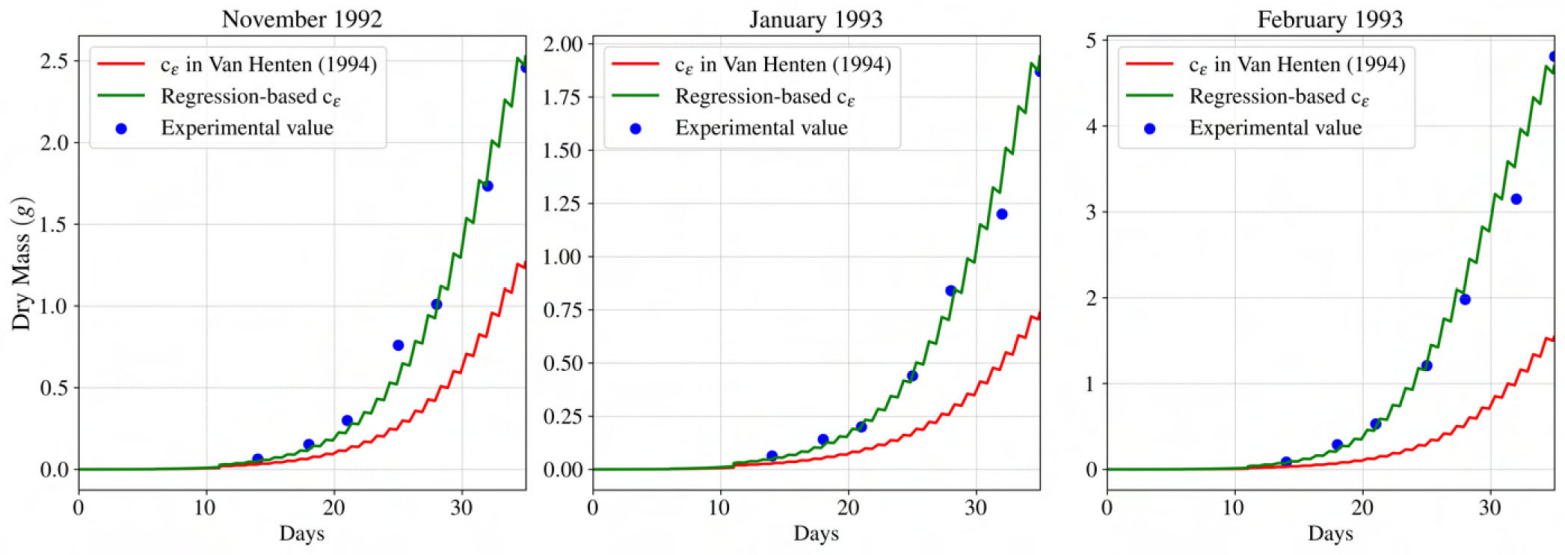
Comparison of dynamic growth model accuracy using the suggested value for c ${\;}_{\varepsilon }$ by Van Henten (1994), and obtained value using the proposed regression-based light-use efficiency model with experimental data from Both et al. (1994) for periods of November 1992, and January and February of 1993. The regression-based model is capable of approximating dry mass for greenhouse cultivated lettuce.

According to **Figure 15**, using the proposed regression-based light-use efficiency model, lettuce dry mass predictions were in close agreement (mostly within 2-3 percent error) with the experimental data.

## 4 Discussion and conclusion

The aim of this study is to predict the impact of incoming light spectral distribution and its intensity on lettuce growth. For this purpose, a dynamic model of plant growth for lettuce provided by Van Henten (1994) is modified. An ODE solver is developed to simulate the dynamic behavior of lettuce from the seedling stage to maturity. It is assumed that the spectral distribution of light and its intensity affect the model through a coefficient $c_{\varepsilon }$ , which accounts for energy provided by photons for a reduction of one molecule of CO ${\;}_{2}$ . Using data for lettuce cultivated under 20 different indoor lighting treatments, the ODE solver calculated $c_{\epsilon }$ for different cases. Several models are fitted using spectral distribution ratios for 4 light wavebands: blue (400−500 nm) , green (500−600 nm) , red (600−700 nm) , and far-red (700−750 nm) as input data and the obtained $c_{\varepsilon }$ as the sole output. To determine the algebraic structure of the model with the highest accuracy, a variety of regression models with varying numbers of features, from 4 (ratios for the blue, green, red, and far-red wavebands) to 16 (first, second, third, and fourth-order values for these ratios) are investigated. The combination of first to fourth-order terms that had the highest accuracy (98 %) was a regression model based on integrated spectral irradiance distribution (in which the predicted $c_{\varepsilon }$ was based on normalized overall spectral irradiance). In order to obtain coefficients for different terms in the regression model, 17 of the 20 experimental data were utilized, while the rest prevented the overfitting of the regression model. To further evaluate the accuracy of the regression model, 21 experimental data for three replications of indoor-cultivated lettuce were used (Both et al., 1994) and are presented in **Figure 15**.

**Figure 16:**
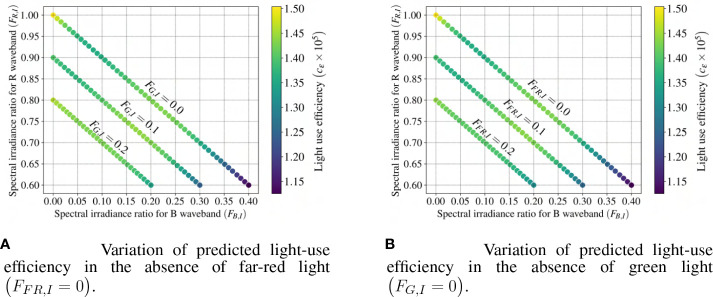
Impact of the incoming light spectral distribution on the light-use efficiency coefficient ( $c_{\varepsilon }$ ) considering the same spectral irradiance integrated over wavelength (IPAR+F R remains unchanged). B and R refer to blue and red. The constraint of Equation (13) enforces a linear relationship between FB,I and FR,I within each scenarios, and ensures a clear visualization of the effect of the spectral distribution on $c_{\varepsilon }$ .

The impact of incoming spectral distribution on lettuce plant growth is investigated. By considering two constrained scenarios, it is possible to visualize the impact of varying spectral distribution on light-use efficiency. In these scenarios, the spectral irradiance integrated over wavelengths (I_PAR+FR_) remains unchanged while one of the wavebands is eliminated from the spectra. In the first scenario, it is assumed that the far-red waveband is missing from the light spectra and only contains the traditionally defined PAR waveband. Contrary to the first scenario, in the second one, the green waveband is replaced with far-red; thus, the incoming spectrum is composed of blue, red, and far-red wavebands. **Figures 16A, B** demonstrate how light-use efficiency varies in scenarios one and two, respectively. Based on these figures, the red waveband promotes and the blue waveband inhibits biomass accumulation in lettuce by increasing and decreasing light-use efficiency, respectively. The impact of the blue waveband on plant growth is enhanced by the presence of the green waveband. On the other hand, the addition of the far-red waveband to the light spectrum mitigates the impact of the blue/green waveband. Therefore, maximum lettuce biomass accumulation can be achieved using the outcomes of these scenarios by emphasizing the red and far-red wavebands and avoiding a high spectral irradiance ratio of the blue and green bands. However, this model ignores important quality considerations such as leaf color, texture, nutritional content, and post-harvest longevity.

The model presented in Equation (12) provides a simplified framework to evaluate the impact of spectral distribution on lettuce plant growth. This contrasts with a model for tomato growth (Dieleman et al., 2019), in which the 3D model needs to be solved in order to investigate the effect of light quality. Moreover, the application of this model to the cultivation of lettuce can increase biomass accumulation during the plant growth cycle. This model can also be used to optimize light conditions, allowing for more efficient use of energy and resources.

Although the regression model predictions are in good accordance with the solver predictions, additional experimental data with a focus on the impact of light spectrum on lettuce plant morphology will likely create a more comprehensive model with higher fidelity. Furthermore, adding higher-order terms to the regression model resulted in a decrease in the weights of the first-order terms, which in turn suggests the necessity of investigating regression models with higher complexity on more comprehensive data for developing an accurate model of light-use efficiency. Since in the studied dataset, the number of samples is limited to 20 experiments, it is not feasible to assess the performance of more complex regression models. In addition, interactions likely exist between light intensity and the photon spectrum, and additional data are needed to test and improve the model’s performance. Specifically, morphological acclimation, such as total leaf area, canopy area, number of leaves, leaf pigmentation, and chlorophyll concentration, can affect the photosynthetic rate and light interception and would ideally be parameterized in future growth models. Finally, this technique has the potential to be applied to other horticultural crops, particularly leafy vegetable crops, to incorporate the impact of spectral distribution on biomass accumulation and crop yield.

## Data Availability Statement

The data analyzed in this study is subject to the following licenses/restrictions: Data was extracted from published articles cited in the references. Requests to access these datasets should be directed to benard@msu.edu.

## Author contributions

MA: Conceptualization, Methodology, Software, Formal analysis, Investigation, Writing. XT: Methodology, Software, Review, and editing. ES: Investigation, and Review. ER: Review, Editing, Funding acquisition. JK: Review and editing, Funding acquisition. MM: Review and editing. AB: Supervision, Review and editing, Conceptualization, Project administration, and Funding acquisition. All authors contributed to the article and approved the submitted version.

## Footnotes

1 In order to mitigate potential overfitting, the linear regression was also performed using both *ridge* and *LASSO* regularization, again using Scikit-learn built-in function. **Figure 7** indicates the importance of high-order polynomial terms but is not regularized; however, results in other figures employ ridge regression.

## Glossary

**Table:**

$C_{CO_{2}}$	Carbon dioxide concentration $\left(mL\;L^{-1}\right)$
*DLI*	Daily light integral $\left(mol\;m^{-2}\;day^{-1}\right)$
*F*	Intensity ratio corresponding to specific wavelengths in the form of $\left(\frac{PFD_{specific}}{PFD_{overall}}\right)$ or $\left(\frac{I_{specific}}{I_{overall}}\right)$
*f*	Mass rate of change $\left(g\;m^{-2}\;s^{-1}\right)$
*g*	Conductance $\left(m\;s^{-1}\right)$
*I*	Spectral irradiance integrated over wavelengths $\left(W\;m^{-2}\right)$
*PFD*	Photon flux density $\left(\mu mol\;m^{-2}\;s^{-1}\right)$
$R_{I}$	Ratio of spectral irradiance integrated over PAR+FR wavebands to a spectral irradiance reference value (R180 is considered as the reference) $\left(\frac{I_{PAR+FR}}{I_{PAR+FR,R180}}\right)$
$r_{gr}$	Growth rate of structural material $\left(g\;m^{-2}\;s^{-1}\right)$
*SPFD*	Spectral photon flux density $\left(\mu mol\;m^{-2}\;s^{-1}\;nm^{-1}\right)$
*T*	Temperature $\left({\;}^{\circ }C\right)$
*X*	Dry Mass $\left(g\;m^{-2}\right)$
Subscripts:	
*B*	Blue light waveband (400−500 nm)
*bnd*	Boundary layer
*car*	Carboxylation
*FR*	Far-red light waveband (700−750 nm)
*G*	Green light waveband (500−600 nm)
*K*	Extinction coefficient
*lar*	Leaf area ratio
*max*	Saturation rate $\left(g\;m^{-2}\;s^{-1}\right)$
*nsdm*	Non-structural dry mass $\left(g\;m^{-2}\right)$
*PAR*	Photosynthetically active radiation (400−700 nm)
*PPFD*	Photosynthetic photon flux density $\left(\mu mol\;m^{-2}\;s^{-1}\right)$
*phot*	Carbon dioxide photosynthesis (gm^–2^ s^–1^)
*Q*_10_	*Q*_10_ factor
*R*	Red light waveband (600 – 700 nm)
*resp*	Maintenance respiration (gm^–2^ s^–1^)
*rt*	Root
*sdm*	Structural dry mass (g m^–2^)
*sht*	Shoot
*stm*	Stomata
Greek Letters:	
α	Conversion of assimilated CO_2_
β	Yield factor
γ	Growth rate coefficient
Γ	CO_2_ compensation point (ml L^–1^)
ε	Light-use efficiency $\left(g\;J^{-1}\right)$
τ	Ratio of root dry mass to total dry mass
ω	Density of CO ${\;}_{2}$ $\left(g\;m^{-3}\right)$
